# The B-Raf Status of Tumor Cells May Be a Significant Determinant of Both Antitumor and Anti-Angiogenic Effects of Pazopanib in Xenograft Tumor Models

**DOI:** 10.1371/journal.pone.0025625

**Published:** 2011-10-05

**Authors:** Brunilde Gril, Diane Palmieri, Yong Qian, Talha Anwar, Lilia Ileva, Marcelino Bernardo, Peter Choyke, David J. Liewehr, Seth M. Steinberg, Patricia S. Steeg

**Affiliations:** 1 Women's Cancers Section, Laboratory of Molecular Pharmacology, Center for Cancer Research, National Cancer Institute, Bethesda, Maryland, United States of America; 2 Laboratory Animal Science Program, Science Applications International Corporation-Frederick, National Cancer Institute- Frederick, Frederick, Maryland, United States of America; 3 Molecular Imaging Program, Center for Cancer Research, National Cancer Institute, Frederick, Maryland, United States of America; 4 Biostatistics and Data Management Section, Center for Cancer Research, National Cancer Research, National Institutes of Health, Bethesda, Maryland, United States of America; Ohio State University, United States of America

## Abstract

Pazopanib is an FDA approved Vascular Endothelial Growth Factor Receptor inhibitor. We previously reported that it also inhibits tumor cell B-Raf activity in an experimental brain metastatic setting. Here, we determine the effects of different B-Raf genotypes on pazopanib efficacy, in terms of primary tumor growth and anti-angiogenesis. A panel of seven human breast cancer and melanoma cell lines harboring different mutations in the Ras-Raf pathway was implanted orthotopically in mice, and tumor growth, ERK1/2, MEK1/2 and AKT activation, and blood vessel density and permeability were analyzed. Pazopanib was significantly inhibitory to xenografts expressing either exon 11 mutations of B-Raf, or HER2 activated wild type B-Raf; no significant inhibition of a xenograft expressing the common V600E B-Raf mutation was observed. Decreased pMEK staining in the responsive tumors confirmed that B-Raf was targeted by pazopanib. Interestingly, pazopanib inhibition of tumor cell B-Raf also correlated with its anti-angiogenic activity, as quantified by vessel density and area. In conclusion, using pazopanib, tumor B-Raf status was identified as a significant determinant of both tumor growth and angiogenesis.

## Introduction

The validation of drug targets, particularly for multi-kinase inhibitors, will be key to predicting sensitivity and developing rational strategies to address resistance. Pazopanib is an anti-angiogenic drug, binding to the ATP pockets of VEGFR1 (Vascular Endothelial Growth Factor Receptor), VEGFR2, VEGFR3, PDGFRβ (Platelet-Derived Growth Factor Receptor), PDGFRα and c-kit in the low nanomolar range [1]. Its anti-angiogenic activity was observed using corneal micropocket and matrigel plug assays. Anti-tumor activity was demonstrated in various human tumor xenografts [1]. In 2009 pazopanib was approved by the FDA (Food and Drug Administration) for the treatment of advanced renal cell carcinoma.

We recently identified B-Raf as a new, direct target for pazopanib [2]. Pazopanib altered the in vitro signaling of a brain metastatic derivative of MDA-MB-231 breast carcinoma cells, 231-BR, resulting in a reduction in the activity of the ERK pathway despite the presence of both Ras and B-Raf mutations. Enzymatic assays showed direct inhibition of B-Raf by pazopanib. In vivo, pazopanib prevented experimental brain metastases by 231-BR cells or HER2 transfectants of MCF7 breast carcinoma cells (selected for brain tropism, (MCF7-HER2-BR3)) by 73% and 55%, respectively; a concomitant reduction in pERK activity was observed, suggesting that B-Raf was a drug target in vivo. No anti-angiogenic response was observed in the brain metastasis models, which may reflect the highly vascular nature of the brain where co-option of existing blood vessels is predicted to occur [3], [4], [5].

B-Raf is a serine/threonine kinase responsible for the activation of the MEK-ERK signaling pathway downstream of the Ras GTPase. Both Ras and Raf are gene families with multiple interactions among members resulting in complex signaling [6]. Numerous drugs have been developed to target Raf, in particular B-Raf activated by a V600E mutation common in melanoma [7], [8], [9], [10]. A series of recent reports extensively studied the complex mechanisms of action of several Raf inhibitors such as Sorafenib, PLX4032 and PLX4720 [7], [8], [9], [11], [12], [13]. These reports demonstrate potential adverse effects of Raf inhibitors depending on the tumor genotype, such as the paradoxical activation of C-Raf and the downstream MEK-ERK pathway in tumor cells expressing mutant Ras.

The effect of pazopanib on the spectrum of B-Raf mutations remains to be determined, as well as the relative contributions of its various targets to its anti-tumor effects. In the current report, a panel of seven breast carcinoma and melanoma tumor cell lines was used to further define the spectrum of pazopanib activity both in vitro and in vivo. The data point to a unique pattern of in vivo selective activity for pazopanib relative to B-Raf signaling. The data also identify a previously unrecognized association between tumor cell B-Raf status and anti-angiogenic activity in vivo.

## Materials and Methods

### Drugs and cell lines

Pazopanib was provided by GlaxoSmithKline. Pazopanib powder was reconstituted in DMSO and stored at −80°C (20 mM stock). For in vivo experiments the vehicle was 0.5% hydroxypropylmethylcellulose with 0.1% Tween 80 in water. The human MDA-MB-231 BR “brain seeking” (231-BR) cell line and its culture were previously described [14], [15]. MCF7 and MCF7-HER2 (HER2 accession number: NM004448) were kindly provided by Dr. Dennis Slamon (University of California Los Angeles, Los Angeles, CA, USA) and maintained in RPMI-1640 (Invitrogen) supplemented with 10% FBS and 1% penicillin-streptomycin solution. SKMEL2 and SKMEL28 were kindly provided by Dr. Michael Gottesman (National Cancer Institute, NIH, Bethesda, MD) and maintained in RPMI supplemented with 10% FBS, penicillin-streptomycin solution and 2 mM glutamine (Invitrogen). WM3899 and WM3918 melanoma cell lines, isolated from patients, were provided by Dr. Meenhard Herlyn (The Wistar Institute of Anatomy and Biology, Philadelphia, PA). These cell lines were maintained in Tu2% growth media (80% MCDB153, 20% Leibovitz's L-15, 2% FBS, 5 µg/ml Bovine Insulin and 1.68 mM CaCl2).

### In vitro experiments

Standard procedures were used for immunoblot analysis and viability assays, which were described previously [2]. For the immunoblot analysis, total and pERK1/2, PDGFRα and β, B-Raf, VEGFR3 and c-kit antibodies were obtained from Cell Signaling Technology and used at a 1∶1000 dilution. PlGF (Placenta Growth Factor), VEGF, and VEGFR1 antibodies were obtained from Santa Cruz Biotechnology and used at 1∶500. The exposure time for the western blots was optimized to avoid saturation of the strongest signals. The B-Raf kinase assay is described in the Material and Methods S1. For B-Raf siRNA transfection, siRNA constructs were purchase as Duplexed Stealth™ RNAi (Invitrogen). The protocol and siRNA sequences were described previously [2]. The protocol for B-Raf siRNA transfection is described in the Material and Methods S2. For the cell viability assay, cell lines were plated at a density of 5,000 cells/well in 24-well plates and incubated overnight to allow cells to adhere. Tumor cell lines were maintained in 10% FBS and treated with increasing concentrations of pazopanib (1–10 µM) or with DMSO as a control, for 96 hours. The number of viable cells was determined by counting, with a hemocytometer, trypsinized cells that excluded trypan blue dye. A second method was also performed, using 3-(4,5-dimethyl-2-thiazolyl)-2,5-diphenyl-2H-tetrazolium bromide (MTT; Sigma) in a 96-well plate format. Results are representative of three independent experiments, each performed in triplicate or quintuplicate.

### Primary tumor formation

Animal experiments were conducted under an approved Animal Use Agreement with the NCI. For the breast cancer cell lines (231-BR, MCF7 and MCF7-HER2), 5- to 7-week-old female athymic nude mice (nu/nu) (Charles River Laboratories) were anesthetized under isoflurane/O_2_ and 5×10^6^ cells were inoculated in the fourth mammary fat pad. One day before MCF7 and MCF7-HER2 implantation then, once a week thereafter, mice were injected with 1.5 mg/kg 17 β-estradiol (Innovative Research of America) to promote tumor cell growth. There were two independent experiments performed on the breast cancer cell lines (representative results are presented). For the melanoma cell lines (SKMEL28, SKMEL2, WM3899 and WM3918), 5- to 7-week-old female athymic nude mice (nu/nu) were injected subcutaneously into one flank. Cells were injected in 100 µL of matrigel (BD Biosciences) (WM3899: 500,000 cells per mouse, WM3918: 1×10^6^, SKMEL28: 5×10^6^, SKMEL2: 5×10^6^).

For each experiment, 7–10 mice were used in each treatment group. Treatment started when the average tumor size was approximately 100 mm^3^ and was administered by oral gavage twice daily. During treatment, measurements of the tumor size and mouse weight were calculated twice weekly. For tumor volume determination: Two-dimensional measurements were taken twice per week with a caliper and tumor volume (V) calculated using the following formula: V = a×b^2^×0.52, where “a” is the longest diameter, “b” is the shortest one, and 0.52 is a constant to calculate the volume of an ellipsoid.

### Immunohistochemistry and Immunofluoresence

The protocols were described previously [2]. For each primary tumor, positively stained cells for pERK1/2, pMEK1/2 and pAKT were counted at 400× magnification in three different “hot-spot” fields per tumor. After CD31 staining, the number of blood vessels and percentage of area covered by blood vessel were measured at 100× magnification in three different “hot-spot” fields per tumor.

### In Vivo DCE-MRI

DCE-MRI (Dynamic contrast enhanced-magnetic resonance imaging) was performed by taking a series of 60 3D T1-weighted FFE dynamic images (TR = 11 ms, TE = 2.3 ms, matrix = 512×512, FOV = 80×80 mm, slices = 18, thickness = 1 mm, scan time = 45 sec) in the coronal plane. Magnevist (Bayer HealthCare Pharmaceuticals,Wayne, NJ) was administrated at a concentration of 0.2 mmol Gd/kg mouse in the tail IV as a bolus injection (150 µL/min) after the first dynamic scan. The DCE-MRI analyses are described in the Material and Methods S3.

### Statistical analysis

Methods are described in Material and Methods S4.

## Results

### Characterization of the breast cancer and melanoma cell lines used

We previously identified a B-Raf inhibitory activity for the anti-angiogenic drug pazopanib [2]. The activity of pazopanib against the spectrum of B-Raf mutations, and its relationship to other known targets remains unknown. To investigate these questions, a panel of seven human breast carcinoma and melanoma cell lines was examined for pazopanib responsiveness in vitro and in vivo. Table 1 lists the seven cell lines, their reported Raf pathway mutational status, and their expression of relevant pazopanib pathway components, determined by western blot (data not shown). The breast cancer cell lines included the brain seeking variant of the “triple negative” MDA-MB-231 cell line, 231-BR, the estrogen receptor positive MCF7 cell line and the MCF7 line transfected with HER2. The melanoma cell lines included the WM3899 cell line, the WM3918 line, the SKMEL28 line and the SKMEL2 line. As shown in Table 1, these cell lines harbor different phenotypes in regard to both their B-Raf status (wild-type vs. various mutations), and in regard to expression of the number and level of other established pazopanib targets (PDGFRβ/α, VEGFR1/3 and c-kit).

**Table 1:** In vitro growth inhibition by pazopanib on breast carcinoma and melanoma cell lines expressing different genotypes in the Ras-Raf-ERK1/2 pathway*.

				Pazopanib targets∥				
Cell Lines	B-Raf Status	Ras Status	Pazopanib IC50 (µM)†	V1§	V3	Pα	Pα	c-kit
231-BR	G464V	G13D (K-Ras)	1.22	+	−	+	−	−
MCF7-HER2	WT	WT	3.96	+/−	−	−	−	−
MCF7	WT	WT	6.29	+/−	−	−	−	−
WM3899	G469V	WT	2.18	−	+/−	−	−	−
WM3918	WT	WT	2.13	+	+/−	+	−	−
SKMEL28	V600E	WT	6.13	−	+	−	−	−
SKMEL2	WT	Q61R (N-Ras)	5.13	+/−	+/−	−	−	−

*Genotypes for MDA-MB-231 and MCF7 [47]; genotypes for WM3899 and WM3918 personal communication by Dr. Meenhard Herlyn; genotypes for SKMEL2 and SKMEL28 [48].

† IC_50_ measured using a trypan blue exclusion cell viability assay 96 h after pazopanib treatment.

∥ Determined by western blot analysis. (+) when a clear band appeared in less than one minute of exposure; (−) when no signal at all appeared after more than 30 min of exposure; (+/−) when a faint band was observed, after 20 min of exposure.

§ Pα, PDGFRα; Pβ, PDGFRβ; V1, VEGFR1; V3, VEGFR3; WT, Wild Type.

### Pazopanib inhibition of B-Raf is associated with its anti-growth activity in vitro

**Figure 1:**
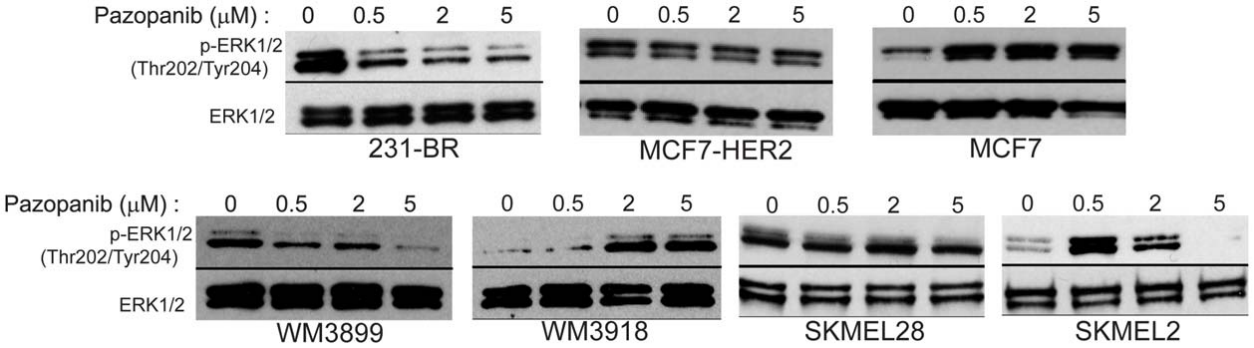
Effect of pazopanib on ERK activation. Tumor cells were serum starved overnight and subsequently treated with pazopanib or DMSO for 24 hours. After treatment, cells were stimulated with 10 ng/mL VEGF for 10 minutes, and cell lysates were analyzed by immunoblot for pERK1/2. The data shown are representative of two conducted experiments.

Pazopanib was tested for inhibition of tumor cell viability. Two independent assays were performed to determine the IC_50_ of pazopanib on each cell line: a trypan blue exclusion assay (Table 1) and an MTT assay (Table S1), with concordant trends. To determine if the B-Raf pathway was modulated by pazopanib, each cell line was treated with vehicle or 0.5–5 µM pazopanib and the pERK1/2 status determined by western blot (Fig. 1). To eliminate the effect of a potential feedback loop mechanism from occurring in cells and altering pERK1/2 levels, a B-Raf kinase assay was also performed on lysates from each cell line (Fig. S1). The cell lines most sensitive to growth inhibition by pazopanib were 231-BR, WM3899 and WM3918 lines, with an IC_50_ of 1.22–2.18 µM (Table 1). Both 231-BR and WM3899 harbor a mutation in exon 11 of B-Raf and were the only cell lines to show a clear decrease in pERK1/2 at all three concentrations of pazopanib (Fig. 1) and a decrease in pMEK1 in the lysate kinase assay (Fig. S1). The other pazopanib sensitive cell line, WM3918, expresses a wild type Ras-Raf-ERK1/2 pathway; however, it also expresses three of the previously established pazopanib targets (VEGFR1, VEGFR3 and PDGFRβ). Expression of these targets potentially accounts for its sensitivity to pazopanib. When the WM3918 cell line was treated with pazopanib an increase in pERK1/2 was detected at the highest concentration used (Fig. 1). Furthermore the kinase assay for this cell line showed no inhibition of pMEK1 (Fig. S1), indicating that pazopanib growth inhibition was independent of B-Raf.

HER2 overexpression by the MCF7 cells, which constitutively activates the B-Raf pathway, altered pazopanib sensitivity. When HER2 was over-expressed in MCF7 cells, the IC_50_ decreased from 6.29 to 3.96 µM. In parental MCF7 cells, pazopanib increased pERK1/2 expression. However the transfection of HER2 into this cell line changed the dynamics of the pathway as shown by a slight decrease in pERK1/2 after pazopanib treatment (Fig. 1), and a slight decrease in pMEK1 in the kinase assay (Fig. S1), concordant with growth inhibition.

The two cell lines which were the least sensitive to the growth inhibitory effect of pazopanib, were the melanoma cell lines: SKMEL2 that expresses mutant N-Ras and the SKMEL28 line that harbors the V600E mutation for B-Raf (IC_50_ 5.13 and 6.13 µM, respectively). Pazopanib had no effect on pERK1/2 in the SKMEL28 cell line (Fig. 1) nor on pMEK1 in cell lysate (Fig. S1). In the SKMEL2 cell line, pazopanib increased pERK1/2 expression at a low concentration but shut down ERK1/2 activation at a higher dose (Fig. 1); no effect of pazopanib was observed on pMEK1 level in the kinase assay (Fig. S1).

Taken together, the data show two-to- three-fold preferential growth inhibition by pazopanib for tumor cell lines harboring mutations in exon 11 of B-Raf or where HER2 activated wild type B-Raf. Growth inhibition was associated with reduced ERK1/2 phosphorylation and MEK1 phosphorylation in the two cell lines harboring mutations in exon 11 of B-Raf. A lesser degree of growth inhibition was observed for MCF7 cells transfected with HER2, which was accompanied by a less pronounced decrease in pERK1/2 and pMEK1 expression. The combination of pazopanib targets, as evidenced in the WM3918 cell line (expressing VEGFR1, VEGFR3 and PDGFRβ), resulted in growth inhibition independent of B-Raf.

To further investigate the correlation between B-Raf status and pazopanib efficacy, 231-BR cells were transfected with siRNA against B-Raf. At 48 h after siRNA transfection, which corresponded to the time point when pazopanib treatment was started, the percentage decrease in B-Raf protein expression for the three experiments conducted was 23–46% and 65–74%, for constructs #1 and #2, respectively. At 144 h after siRNA, which corresponded to 96 h of pazopanib treatment, B-Raf protein knockdown was less pronounced, 3 to 58% for construct #1 and 76 to 89% for construct #2 (Fig. S2 A–B). After 96 h of pazopanib exposure, an MTT assay was performed on the control and B-Raf knockdown transfectants. The B-Raf siRNA construct #2 produced a significant 48% decrease in cell viability compared to cells transfected by the non targeted siRNA (p = 0.010). However, no change in cell viability was observed for construct #1 (Fig. S2C), likely due to insufficient knock down of B-Raf. The two B-Raf siRNA constructs also produced different cellular responses after pazopanib treatment. A significant decrease in the pazopanib IC_50_ was observed in the cells transfected with construct #2 compared to the controls (3.85 compared to 4.9 µM, respectively (p = 0.024)). However, in cells transfected with construct #1, B-Raf was re-expressed after pazopanib treatment, which likely resulted in lesser sensitivity to pazopanib compared to the control (IC_50_ of 6.87 compared to 4.9 µM, respectively (p = 0.008)). A representative experiment is shown on Figure S2D. Overall, the significant differences in pazopanib sensitivity contingent on the level of B-Raf expression confirmed a key role of B-Raf protein in pazopanib's mechanism of action.

### Pazopanib inhibition of primary tumor growth

**Figure 2:**
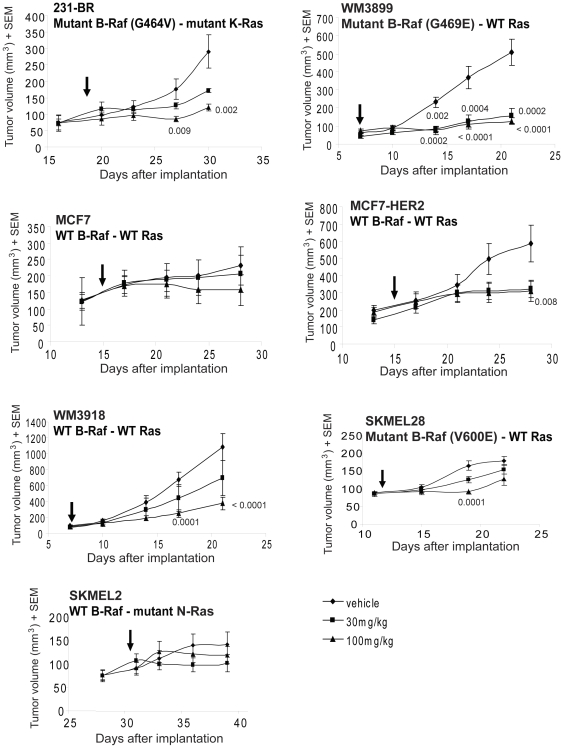
Effect of pazopanib on primary tumor growth of breast cancer and melanoma cell lines. Breast cancer lines were implanted in the mammary fat pad, while melanoma cells were implanted subcutaneously. Mice were randomly chosen to receive vehicle or 30 or 100 mg/kg pazopanib, twice daily for 10–14 days. Tumor size was measured twice weekly. Arrows indicate when treatment started. P values are shown for the tumors in which the decrease in tumor size achieved significance (p<0.01) at a given dose of pazopanib. The results shown for 231-BR, MCF7 and MCF7-HER2 cell lines are representative of two experiments. Raw data means and SEM are presented; analysis was performed on cubed root transformed data.

Primary tumor growth experiments were conducted to determine if B-Raf status also predicted in vivo sensitivity to pazopanib. It was also of interest to determine if the two- to three-fold differences in IC_50_ observed in vitro translated in to significant trends in vivo. The breast cancer cell lines listed in Table 1 were implanted in the mammary fat pad, and the four melanoma cell lines were injected subcutaneously. Mice were randomly chosen to receive vehicle, 30 mg/kg or 100 mg/kg pazopanib, twice daily by oral gavage, once tumors reached a mean of approximately 100 mm^3^ (Fig. 2). In brief, the order of sensitivity of tumor growth inhibition by pazopanib was 231-BR, WM3899, WM3918>MCF7-HER2≫SKMEL2, SKMEL28, MCF7. This trend is comparable to the in vitro sensitivity of each line to pazopanib.

Primary tumor growth produced by the 231-BR cell line, which contains an exon 11 mutation of B-Raf, was maximally sensitive to pazopanib in vitro and significantly inhibited by pazopanib 9 days after treatment with the highest drug dose (p = 0.009). Three days later, the 30 and 100 mg/kg doses induced 40% and 59% tumor growth inhibition, respectively (p = 0.030 (this p value represents a strong trend) and p = 0.002, respectively). The WM3899 cell line, which harbors a similar exon 11 B-Raf mutation and was sensitive to pazopanib in vitro, was also highly sensitive to pazopanib in vivo, with 62% and 67% decreases in tumor size 7 days after treatment at 30 and 100 mg/kg drug, respectively (p = 0.002 and p = 0.0002, respectively). Ten days after starting drug treatment, both doses continued to significantly inhibit tumor growth, and at day 14 of treatment the tumor growth inhibition was 69% and 75% at 30 mg/kg and 100 mg/kg, respectively (p = 0.0002 and p<0.0001, respectively).

The MCF7 cell line, which was less sensitive to pazopanib in vitro, exhibited no statistically significant differences in tumor size through 13 days of treatment. For the MCF7-HER2 cell line, which was 37% more sensitive to pazopanib in vitro, an inhibition of tumor growth at the highest drug dose was observed 9 days after starting treatment (p = 0.042 (this p value represents a strong trend)); both doses were inhibitory 4 days later with 45% and 47% tumor growth inhibition at 30 and 100 mg/kg, respectively (p = 0.019 (this p value represents a strong trend) p = 0.008, respectively).

The WM3918 cell line was significantly inhibited by the highest dose of pazopanib 10 days after treatment (p = 0.0001). Four days later, the highest dose still significantly inhibited the tumor growth (65% of tumor inhibition, p<0.0001). This was the only cell line that expressed three of the established targets of pazopanib, which provides an explanation for its sensitivity to the drug despite the expression of a normal B-Raf pathway.

In contrast, tumor cell lines with mutations shown to be less sensitive to pazopanib inhibition in vitro exhibited less sensitivity to the drug in vivo. For the SKMEL28 melanoma cell line (V600E B-Raf mutation), a modest but significant decrease in tumor size was observed after nine days of treatment, however, none of the doses maintained significant efficacy four days later. For the SKMEL2 cell line, harboring an N-Ras mutation, no efficacy was observed. In summary, the trends in growth inhibition by pazopanib in cell culture were directly correlated with, and magnified in, tumorigenesis in vivo.

### Pazopanib inhibition of the B-Raf pathway in vivo

**Figure 3:**
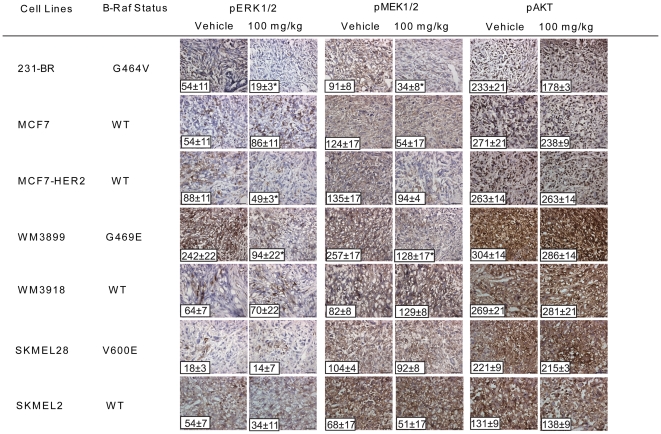
Pazopanib inhibition of primary tumor growth correlates with B-Raf inhibition. Tumor cell lines and the B-Raf genotypes are listed. Representative photographs of pERK1/2, pMEK1/2 and pAKT immunostaining are shown for the vehicle and the 100 mg/kg pazopanib treated groups. The mean number of positive cells ± SEM is shown on each representative image. Asterisks (*) indicate a statistically significant difference between the pazopanib and vehicle treated group (p<0.01). (See Figure S3 for the 30 mg/kg dose data).

To confirm that B-Raf was targeted in the pazopanib-sensitive primary tumors, the phosphorylation levels of the B-Raf downstream targets MEK1/2 and ERK1/2 was determined by immunohistochemistry (Fig. 3 and Fig. S3). For each cell line xenograft, one tissue slide per tumor and five mice per treatment group (vehicle, 30 and 100 mg/kg) were stained for pMEK1/2 and pERK1/2. In each slide the number of positive cells was quantified, with the mean for the five mice reported on Figure 3 at the bottom right of each representative photograph.

In general, cell lines with exon 11 B-Raf mutations or HER2 activation of wild type B-Raf showed evidence of Raf pathway inhibition in vivo. A 44–65% decrease in pERK1/2 staining and a 30–63% decrease in pMEK1/2 were observed in the pazopanib sensitive 231-BR, MCF7-HER2 and WM3899 tumors treated with 100 mg/kg drug, with a trend at 30 mg/kg. The effect of pazopanib on the tumor growth of the WM3918 cell line was confirmed to not be associated with B-Raf targeting as a significant decrease of pMEK1/2 and pERK1/2 was not observed at any drug dose. The inhibition in tumor growth was therefore probably due to the inhibition of the three established targets (VEGFR1, VEGFR3, and PDGFRβ), leading to the inactivation of alternative pathways to the ERK pathway and, subsequently, to a decrease in tumor growth. No activation of ERK1/2 was observed in vivo, as was seen in vitro on Figure 1, suggesting the potential role of the microenvironment in vivo. In contrast, a significant decrease in pMEK1/2 was observed in MCF7 tumors treated with 30 mg/kg pazopanib, but it was not accompanied by a similar decrease in pERK1/2 staining. The remaining tumors did not show any statistically significant decrease in the number of positively stained cells in the 100 mg/kg treatment group for either of the two markers. None of the tumors exhibited decreased pAKT at any drug dose tested (Fig. 3 and Fig. S3). This trend supports the selectivity of pazopanib for the B-Raf pathway in vivo.

### The anti-angiogenic activity of pazopanib is associated with tumor cell B-Raf inhibition

**Figure 4:**
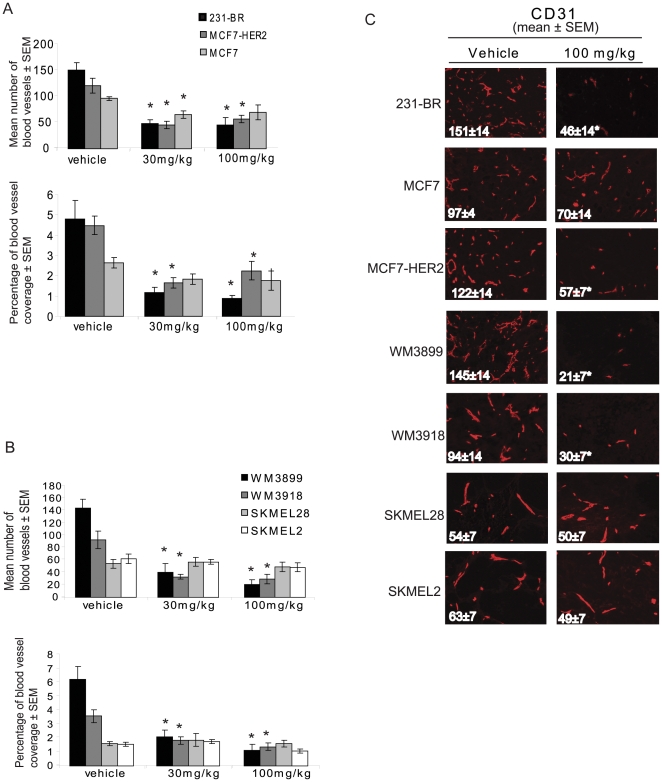
Anti-angiogenic effect of pazopanib on primary tumor xenografts correlates with B-Raf status. **Panel A:** Breast cancer xenografts. **Panel B-** Melanoma xenografts. In each panel the top graph represents the mean number of blood vessels per tumor section (n = 5 mice) ± SEM and the bottom graph represents the percentage of area covered by blood vessels (n = 5 mice) ± SEM for the vehicle treated group, the 30 mg/kg group and the 100 mg/kg group. **Panel C:** Representative photographs of CD31 staining for vehicle and 100 mg/kg treatment groups. Asterisks (*) indicate a statistically significant difference between the pazopanib and vehicle treated groups (p<0.01). (See Figure S4 for representative photographs of CD31 staining).

Pazopanib is well characterized as an anti-angiogenic drug. Normal endothelial cells express VEGFR2 and should have a wild type B-Raf pathway. We therefore hypothesized that pazopanib would exert comparable anti-angiogenic effects on each of the primary tumor xenografts. To test this, measurements of CD31+ vascular density and area of tumor covered by vasculature were determined on the primary tumors (Fig. 4A–B and Fig. S4). Figure 4C and Figure S4 present representative photographs of CD31 staining.

Interestingly, a significant decrease in blood vessel density and percentage of area covered by blood vessels was observed in the pazopanib-sensitive tumors: 231-BR, MCF7-HER2 and WM3899 (p<0.01). Again, these represent the cell lines with either an exon 11 B-Raf mutation or HER2 activation of wild type B-Raf. An anti-angiogenic effect of pazopanib was also observed in the WM3918 tumor. This cell line harbors a normal B-Raf pathway; however, it remained sensitive to pazopanib as shown by the decrease in blood vessel density, probably due to the expression of three of the established targets of pazopanib, leading to the disruption of downstream pathways other than ERK1/2. A significant decrease in blood vessel density was observed in the MCF7 tumor treated with 30 mg/kg but no significant difference was observed in the percentage of blood vessel coverage. Nor was the blood vessel density decrease maintained at the 100 mg/kg dose. No decrease in blood vessel density or percentage of area covered by blood vessels was observed in the SKMEL28 or SKMEL2 cell lines.

**Figure 5:**
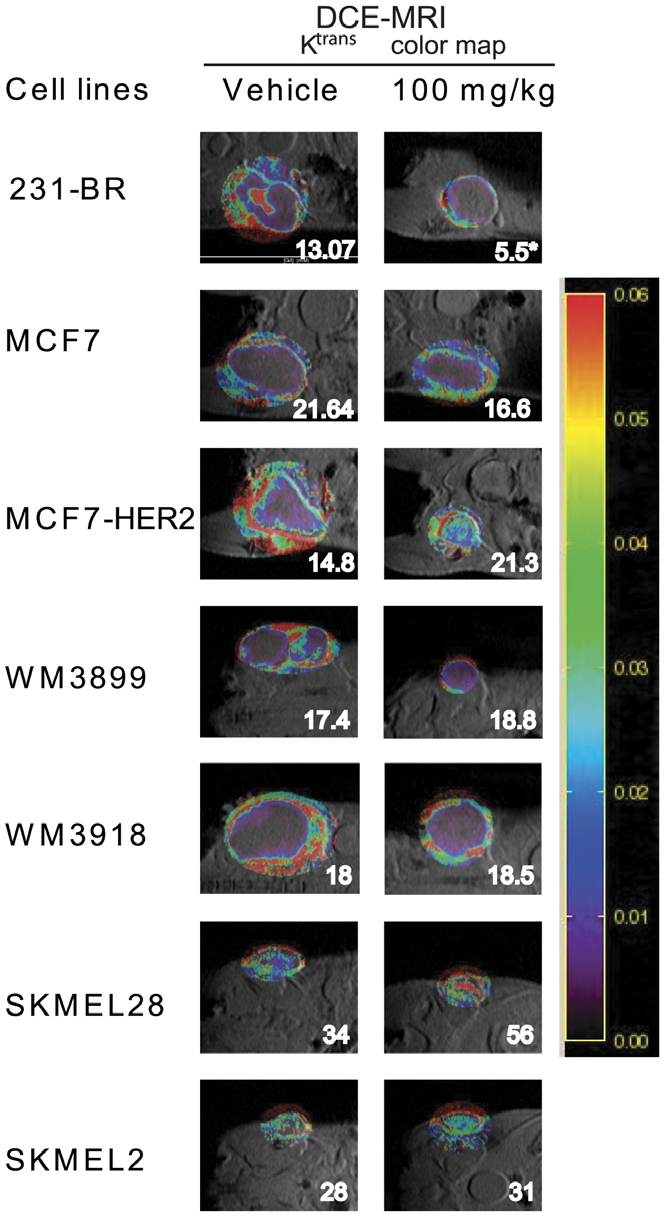
Effect of pazopanib on blood vessel permeability of breast and melanoma cell xenografts. Dynamic Contrast- Enhanced Magnetic Resonance Imaging (DCE-MRI) was performed on 5 mice per group at the end point. Representative photographs and K^trans^ calculations are presented.

To further explore this unexpected trend, another aspect of VEGFR activity was examined, blood vessel permeability. Primary tumors were imaged by DCE-MRI. Indeed, blood vessels in tumors were described as structurally and functionally abnormal [16]. They are more dilated, tortuous and more permeable than normal blood vessels. VEGF, the best known angiogenic molecule, induces survival and proliferation of endothelial cells and increases vascular permeability [17], [18], [19]. DCE-MRI is a widely used noninvasive quantitative method of investigating vascular structure and function by tracking the pharmacokinetics of injected contrast agents as they pass through the tumor vasculature. Representative photographs and K^trans^ calculations are presented on Figure 5. Parametric color maps generated by analysis of DCE-MRI illustrate that most of the tumor vascularity was found in the periphery. The only cell line that exhibited a decrease in K-trans was the 231-BR cell line (p = 0.027). The remaining cell lines demonstrated smaller changes in the mean K^trans^ values. No significant changes in K_ep_ were noted (data not shown). Thus, the only cell line showing significantly reduced vascular permeability also exhibited an exon 11 B-Raf mutation.

### Potential anti-angiogenic factors downstream B-Raf

The three most sensitive tumor cell lines, evidenced by decrease in tumor growth, blood vessel density and pERK1/2 in response to pazopanib, were the 231-BR, MCF7-HER2, and WM3899 cell lines. These cell lines harbor either B-Raf mutations in exon 11 or HER2 overexpression-activated wild type B-Raf. Based on the association between specific B-Raf status, pERK1/2 decrease and a consistent blood vessel density decrease, we hypothesized that the B-Raf pathway may regulate angiogenic factor production. The 231-BR and MCF7-HER2 cell lines were transfected with two different siRNA constructs against B-Raf, and the expression of two major angiogenic growth factors was analyzed by western blot. As controls, the cell lines were transfected with a scramble RNA or were treated with the transfection agent only (Fig. S5). The two siRNA B-Raf constructs decreased the expression level of B-Raf; however no effect on VEGF expression level was observed; a decrease in PlGF was observed only in the 231-BR cell line. To investigate additional angiogenic factors, a protein array for angiogenic related-proteins (covering 55 angiogenesis related proteins) was performed. The angiogenic protein array did not show any detectable difference between B-Raf knock down and control cell lines. However as previously mentioned, the knock down of B-Raf protein was incomplete and the remaining B-Raf expression level may have been sufficient to maintain the integrity of downstream pathways.

## Discussion

**Figure 6:**
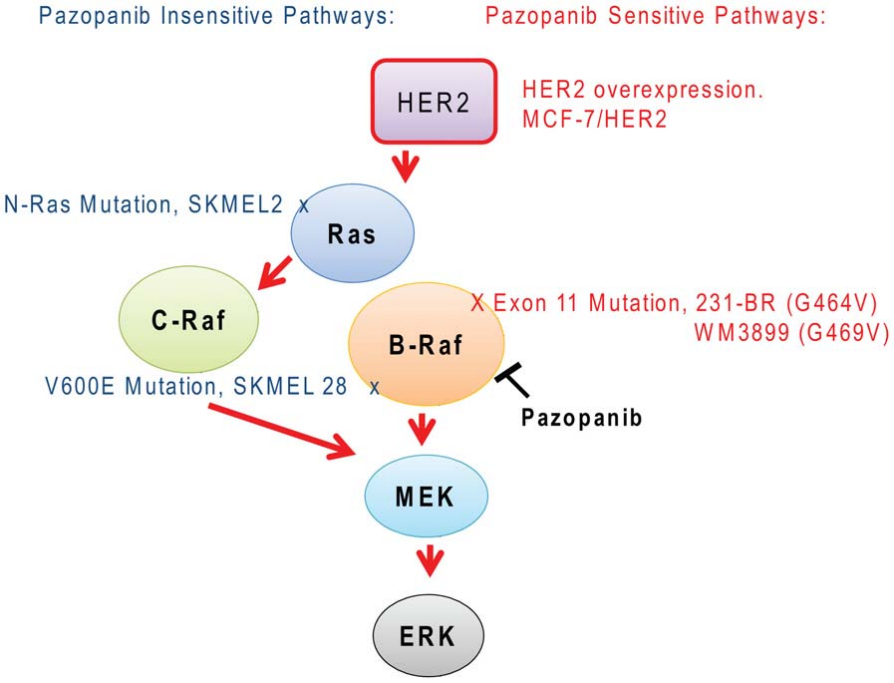
Schematic of pazopanib signaling pathways. The standard receptor tyrosine kinase activation of the ERK pathway is shown. Pazopanib sensitive alterations, including HER2 overexpression and B-Raf exon 11 mutations, are shown on the right in red. Insensitive alterations are shown on the left in blue. Tumor cell lines featuring each alteration are named.

While developed to inhibit VEGFR2 signaling in endothelial cells, pazopanib also inhibits VEGFR1 and 3, PDGFR α and β, and c-kit. Recently a B-Raf inhibitory activity was reported for pazopanib and shown to be operative in its brain metastasis preventative efficacy in two models of HER2 driven breast cancer metastasis to the brain. Herein, we investigated the effects of pazopanib on a panel of seven breast carcinoma and melanoma xenografts to determine the relative contribution of B-Raf versus other targets to its anti-tumor activity (Fig. 6).

Three conclusions are evident from this work. First, pazopanib was directly inhibitory to tumor cells in vitro, in addition to its reported anti-angiogenic effects. These data confirm findings previously reported for lung cancer cells and multiple myeloma [20], [21]. In vitro, two patterns emerged among the seven cell lines for sensitivity to pazopanib: (1) a favorable B-Raf status. B-Raf exon 11 mutations were found in the sensitive 231-BR and WM3899 cell lines while HER2 overexpression occurred in the sensitive MCF7-HER2 cell line, consistent with previously reported enzymatic inhibition assays [2], and; (2) expression of multiple targets, exemplified in the WM3918 cell line expressing PDGFRβ, VEGFR1 and low levels of VEGFR3, but a normal B-Raf. Differences in growth inhibition in vitro were only two-to three-fold, but correlated with magnified, statistically significant inhibition of xenograft growth in vivo. B-Raf inhibition was confirmed in the sensitive cell lines in vitro and in vivo by inhibition of MEK phosphorylation.

Conversely, relative insensitivity to pazopanib was detected in the B-Raf V600E mutated melanoma line SKMEL28 and the SKMEL2 melanoma cell line harboring an N-Ras mutation. In data not shown, a second melanoma cell line expressing mutant N-Ras, WM1366, was also relatively resistant to pazopanib with an IC_50_ of 8 µM. Also, two other melanoma cell lines expressing the V600E B-Raf mutation, WM278 and MALMEL, showed a relatively high IC_50_ similar to SKMEL28 (9 µM); in vivo tests were not conducted. Dumaz et al. reported that when Ras is mutated in melanoma cell lines, tumor cells switch their signaling to C-Raf to activate the MEK/ERK pathway, forgoing B-Raf [22], which may be operative.

Second, the spectrum of pazopanib sensitivity appears to be distinct from other reported Raf inhibitors [7], [8], [9], [11], [12]. The lack of activity towards the common V600E mutated B-Raf suggests limited B-Raf directed activity in melanoma. The exon 11 mutations found in the pazopanib-sensitive 231-BR and WM3899 cell lines are relatively rare in cancer [23], [24]. Pazopanib was significantly more inhibitory to a HER2 transfectant of MCF7 breast carcinoma cells in vivo than the parental cell line, confirming that HER2 activation of wild type B-Raf is a sensitive target for this drug. The B-Raf inhibitor PLX4032 was reported to increase ERK signaling in the HER2+ SKBR3 cell line [9], confirming the differences between these drugs. It will be of interest to determine if overexpression of other receptor tyrosine kinases also confers sensitivity to pazopanib via consistent stimulation of wild type B-Raf.

In cells harboring a wild type genotype in the Ras-Raf pathway, Raf inhibitors induced a paradoxical activation of the downstream MEK-ERK pathway, due to transactivation of C-Raf [7], [8], [9], . However, even if pazopanib induced an increase in the pERK1/2 level in the MCF7 and WM3918 cell lines in vitro, it did not induce any paradoxical activation of the ERK pathway in vivo. These results showed that the mechanism of action of pazopanib appeared to be different from the mechanism of action of other previously studied Raf inhibitors.

We asked whether previous preclinical and clinical research on pazopanib is consistent with the B-Raf pattern observed herein. Pazopanib has been reported to be growth inhibitory in other preclinical xenograft models [1], [21], [25]. It is interesting to note that the least sensitive xenograft in that series was the A375P melanoma, which harbors a V600E mutation in B-Raf [26]. In the clinic, pazopanib produced responses in renal, thyroid, cervical, ovarian and non-small cell lung carcinomas and soft tissue sarcoma [27], [28], [29], [30], [31], [32], [33], [34]. Based on the available data, the V600E mutation is unlikely to be a common driver of B-Raf activation clinically. Of the potential pathway components in the literature, K-Ras mutation, which occurred in 231-BR cells and constitutively activates B-Raf, is found in lung, cervical and ovarian carcinomas [35], [36], [37]; renal carcinomas demonstrate both K-Ras mutation and amplification [38], [39]. HER2 amplification is found in ∼20–25% of breast tumors. Pazopanib has been tested in HER2 overexpressing breast cancer in combination with lapatinib based on the known highly angiogenic nature of these tumors [40], but B-Raf activation may also contribute to the observed efficacy. HER2 amplification is also present in a fraction of other tumor types. Conversely, the clinical literature contains hints that the V600E B-Raf mutation may not confer pazopanib sensitivity. In a radioiodine-refractory metastatic differentiated thyroid cancer cohort, 73% of patients with disease control of ≥1 year had follicular tumors [29]. While V600E B-Raf mutations are common in thyroid cancer, follicular thyroid cancers are negative for this mutation [41]. While fragmentary, the data are consistent with the conclusion that B-Raf activation via the pathways described herein to mediate pazopanib sensitivity may contribute to its clinical efficacy.

Third, the greatest surprise in the present series of experiments was that the anti-angiogenic activity of pazopanib was not equivalent in all xenografts, but correlated with tumor B-Raf pathway sensitivity. Thus, the 231-BR, MCF7-HER2, WM3899 and WM3918 cell lines exhibiting either exon 11 mutations, HER2 overexpression, or multiple pazopanib targets were the only xenografts with a significant anti-angiogenic response to pazopanib. This trend held when angiogenesis was measured by microvessel density or area covered by vasculature. An exception to this conclusion was an analysis of permeability by DCE-MRI, which may reflect the late time point used where only a rim of tumor was analyzed. The data indicate that tumor cell B-Raf plays a significant role in angiogenesis.

The decrease in blood vessel density in response to pazopanib is unlikely to be explained by a simple decrease in tumor size. For example, MCF7 tumors treated with 100 mg/kg pazopanib reached a volume of 200 mm^3^ at the end point with a blood vessel density of 70±14; in the MCF7 transfected with HER2 xenografts similarly treated, tumor size at the end point was larger (300 mm^3^), however the blood vessel density was lower (57±7). Similarly, the WM3899 (B-Raf mutation in the exon 11) and the SKMEL28 tumors were both of comparable size at the experimental end point; however, the blood vessel density in the WM3899 tumor was half of the vessel density in the SKMEL28 tumors (21±7 versus 50±7).

A second hypothesis to explain the data was that the tumor cell B-Raf pathway controlled the production of angiogenic cytokines. The Ras-Raf-MEK-ERK pathway induces VEGF expression directly or indirectly [42], [43], [44], [45], [46]. Herein, knockdown of B-Raf by siRNA in two cell lines failed to consistently modulate VEGF, PlGF or an array of angiogenic related proteins. However, this result may not be sufficient to exclude the involvement of angiogenic pathways downstream B-Raf because the angiogenesis array was performed in vitro and therefore could not mimic the in vivo direct interactions between tumor cells and endothelial cells. Moreover, a better knockdown of B-Raf may be necessary to block potential downstream angiogenic pathways.

Our data suggest that several markers, including B-Raf status (pMEK/pERK), Ras mutation and HER2/receptor tyrosine kinase activation, should be investigated in tumors to determine if they constitute predictive markers of efficacy for pazopanib treatment.

## Supporting Information

Figure S1 **Kinase assay on cell lysates.** Increasing concentrations (0.022, 00.22, 2.2 µM) of pazopanib were incubated with cell lysates for 20 min at 30°C. Inactive MEK1 was added for 30 min and the level of MEK1 phosphorylation was analyzed with pMEK1 and total MEK1 antibodies. The source of the cell lysate is indicated under each panel.
(PDF)

Figure S2 **Cell viability assay on 231-BR cells with siRNA-reduced B-Raf expression.** 231-BR cells were transfected with two different B-Raf siRNA constructs (S1 and S2), with a non targeting siRNA (C), or treated with the transfection agent alone (T). Cells were trypsine and seeded in a 96 h well plates and 6-well plates. At T0, corresponding to 48 h after transfection, cells were treated with increasing concentrations of pazopanib in the 96 well plate and an MTT assay was performed 96 h later (T96). Cell lysates were collected (from plates seeded in parallel) at T0 and T96 to check the level of B-Raf and Tubulin expression. A- Western blot of B-Raf expression at T0 and T96. Data from one of three experiments is shown. B- Percent of densitometric ratio B-Raf/Tubulin compared to the non targeting siRNA control (from panel A). C- Viability at T96 without any pazopanib treatment (average of the three experiments). D- Cell viability by MTT assay at T96 with the indicated Pazopanib treatments.
(PDF)

Figure S3 **pERK1/2, pMEK1/2 and pAKT staining in primary tumors.** For each cell line, pERK1/2, pMEK1/2 and pAKT expression was quantified on five mice per treatment group. Three photographs of “hot spot” staining per section were used to quantify the number of positive cells. P values are shown for the markers that achieved significance at a given dose of pazopanib (P<0.01).
(DOC)

Figure S4 **CD31 staining in primary tumors.** Five mice per group and one section per mouse were stained for CD31. Three photographs of “hot spot” staining were used for quantification. Panels A and B show representative photographs of CD31 staining for each tumor (100× magnification). The AxioVision4 software was used to quantify the number of blood vessels per photograph and the percentage of area occupied by blood vessels, (numbers under each photograph in A and B). The numbers represent the mean number of vessels ± SEM in three “hot spots” per section. P values are shown for the markers that achieved significance at a given dose of pazopanib (P<0.01).
(DOC)

Figure S5 **B-Raf siRNA transfection in the 231-BR and the MCF7-HER2 cell lines.** 231-BR (A) and MCF7-HER2 (B) cell lines were transfected with two different B-Raf siRNA constructs (S1 and S2), with a non targeting siRNA (C), or treated with the transfection agent alone (T). Cell lysates were collected at 48, 72 and 96 h after transfection and analyzed by western blot for B-Raf, Tubulin, PlGF and VEGF expression levels.
(PDF)

Table S1 **In vitro growth inhibition of pazopanib on breast carcinoma and melanoma cell lines by MTT assay.**
(DOC)

Material and Methods S1 **B-Raf kinase assay.**
(DOC)

Material and Methods S2 **B-Raf siRNA transfection followed by cell viability assay.**
(DOC)

Material and Methods S3 **DCE-MRI Analysis.**
(DOC)

Material and Methods S4 **Statistical analysis.**
(DOC)
