# Supplementary material for: The B-Raf Status of Tumor Cells May Be a Significant Determinant of Both Antitumor and Anti-Angiogenic Effects of Pazopanib in Xenograft Tumor Models
Source: PLoS One. 2011 Oct 5;6(10):e25625. doi: 10.1371/journal.pone.0025625 (PMC3187787; doi:10.1371/journal.pone.0025625)
Supplement: Figure S1 — Kinase assay on cell lysates. Increasing concentrations (0.022, 00.22, 2.2 µM) of pazopanib were incubated with cell lysates for 20 min at 30°C. Inactive MEK1 was added for 30 min and the level of MEK1 phosphorylation was analyzed with pMEK1 and total MEK1 antibodies. The source of the cell lysate is indicated under each panel. (PDF) [file pone.0025625.s001.pdf]

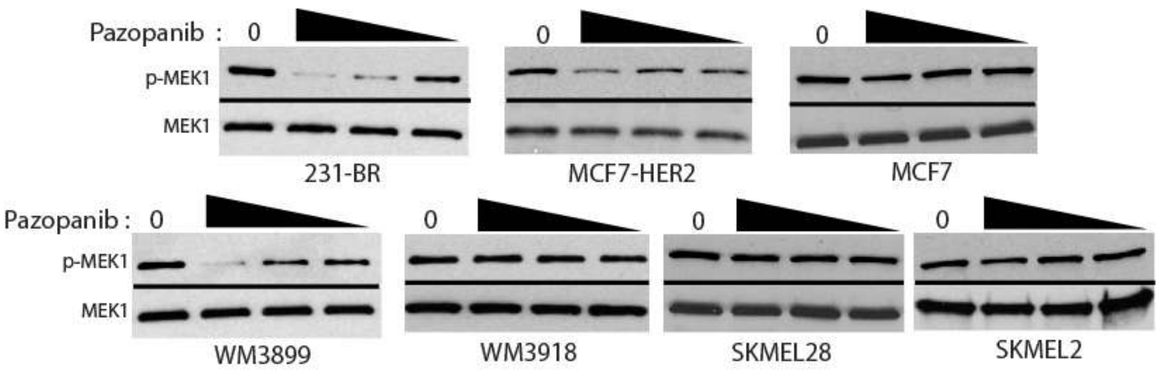

Figure S1. Kinase assay on cell lysates. Increasing concentrations (0.022, 00.22, 2.2 µM) of pazopanib were incubated with cell lysates for 20 min at 30 °C. Inactive MEK1 was added for 30 minutes and the level of MEK1 phosphorylation was analyzed with pMEK1 and total MEK1 antibodies. The source of the cell lysate is indicated under each panel.
