# Supplementary material for: The B-Raf Status of Tumor Cells May Be a Significant Determinant of Both Antitumor and Anti-Angiogenic Effects of Pazopanib in Xenograft Tumor Models
Source: PLoS One. 2011 Oct 5;6(10):e25625. doi: 10.1371/journal.pone.0025625 (PMC3187787; doi:10.1371/journal.pone.0025625)
Supplement: Figure S2 — Cell viability assay on 231-BR cells with siRNA-reduced B-Raf expression. 231-BR cells were transfected with two different B-Raf siRNA constructs (S1 and S2), with a non targeting siRNA (C), or treated with the transfection agent alone (T). Cells were trypsine and seeded in a 96 h well plates and 6-well plates. At T0, corresponding to 48 h after transfection, cells were treated with increasing concentrations of pazopanib in the 96 well plate and an MTT assay was performed 96 h later (T96). Cell lysates were collected (from plates seeded in parallel) at T0 and T96 to check the level of B-Raf and Tubulin expression. A- Western blot of B-Raf expression at T0 and T96. Data from one of three experiments is shown. B- Percent of densitometric ratio B-Raf/Tubulin compared to the non targeting siRNA control (from panel A). C- Viability at T96 without any pazopanib treatment (average of the three experiments). D- Cell viability by MTT assay at T96 with the indicated Pazopanib treatments. (PDF) [file pone.0025625.s002.pdf]

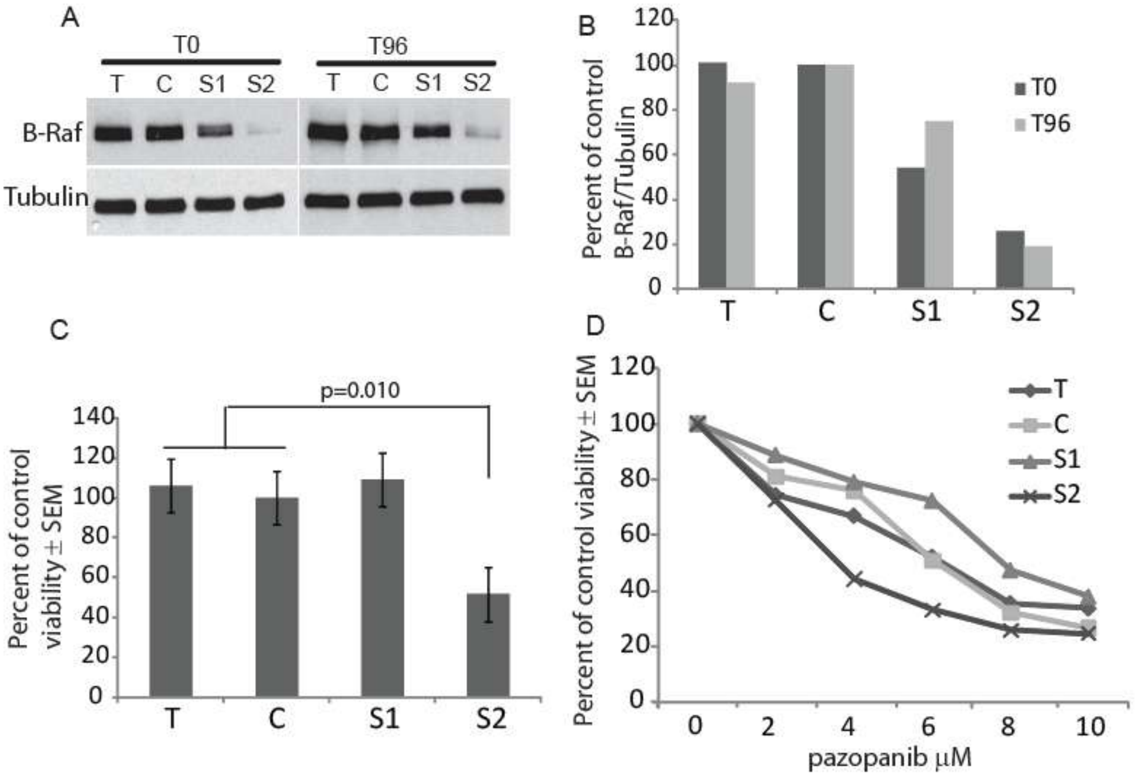

Figure S2. Cell viability assay on 231-BR cells with siRNA-reduced B-Raf expression. 231-BR cells were transfected with two different B-Raf siRNA constructs (S1 and S2), with a non targeting siRNA (C), or treated with the transfection agent alone (T). Cells were trypsine and seeded in a 96h well plates and 6-well plates. At T0, corresponding to 48h after transfection, cells were treated with increasing concentrations of pazopanib in the 96 well plate and an MTT assay was performed 96h later (T96). Cell lysates were collected (from plates seeded in parallel) at T0 and T96 to check the level of B-Raf and Tubulin expression. A- Western blot of B-Raf expression at T0 and T96. Data from one of three experiments is shown. B- Percent of densitometric ratio B-Raf/Tubulin compared to the non targeting siRNA control (from panel A). C- Viability at T96 without any pazopanib treatment (average of the three experiments). D- Cell viability by MTT assay at T96 with the indicated Pazopanib treatments.
