# Supplementary material for: The B-Raf Status of Tumor Cells May Be a Significant Determinant of Both Antitumor and Anti-Angiogenic Effects of Pazopanib in Xenograft Tumor Models
Source: PLoS One. 2011 Oct 5;6(10):e25625. doi: 10.1371/journal.pone.0025625 (PMC3187787; doi:10.1371/journal.pone.0025625)
Supplement: Figure S3 — pERK1/2, pMEK1/2 and pAKT staining in primary tumors. For each cell line, pERK1/2, pMEK1/2 and pAKT expression was quantified on five mice per treatment group. Three photographs of “hot spot” staining per section were used to quantify the number of positive cells. P values are shown for the markers that achieved significance at a given dose of pazopanib (P<0.01). (DOC) [file pone.0025625.s003.doc]

**
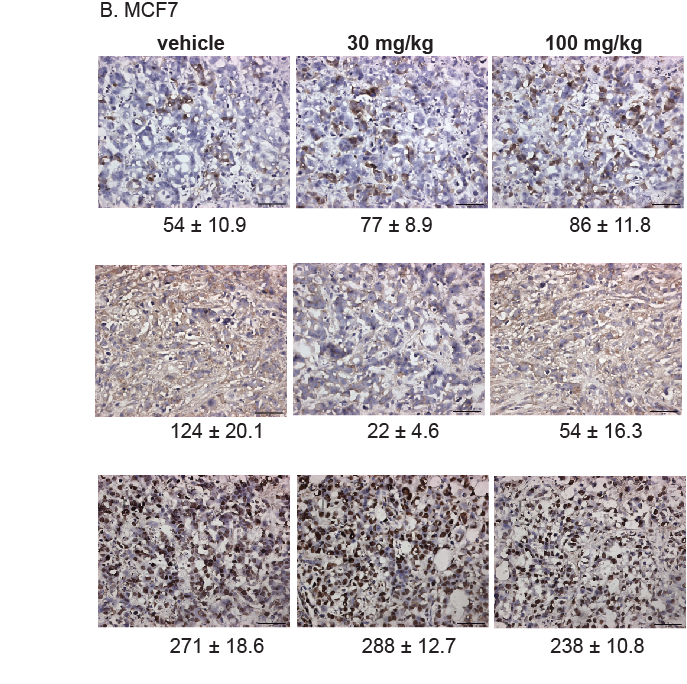

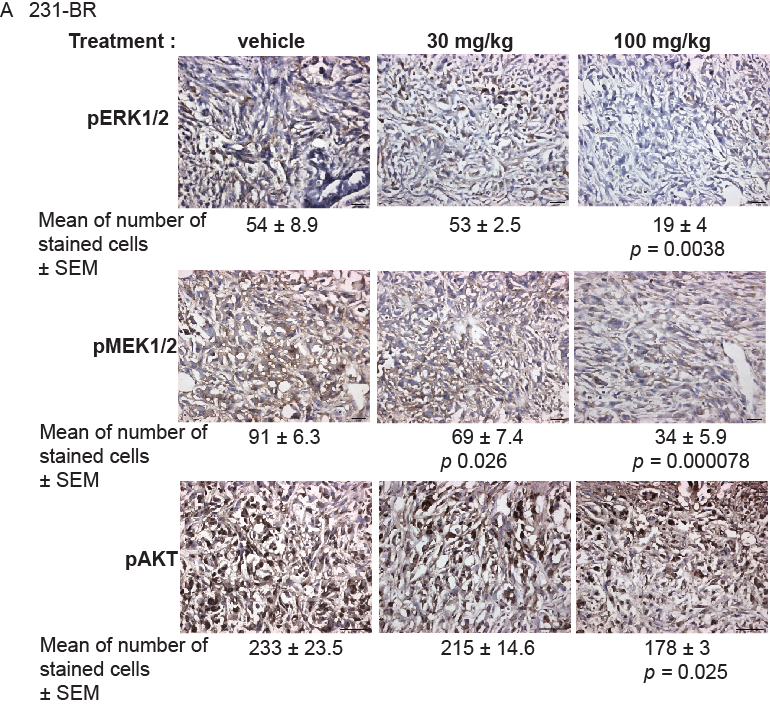

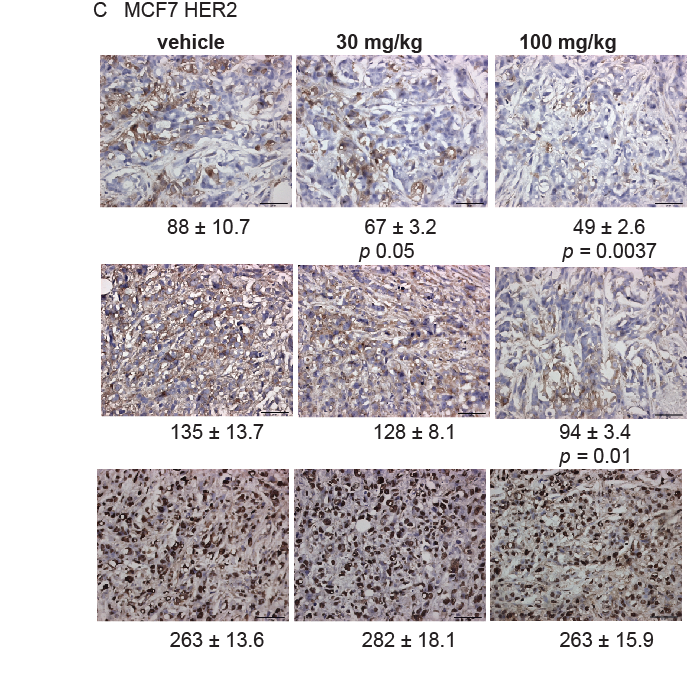

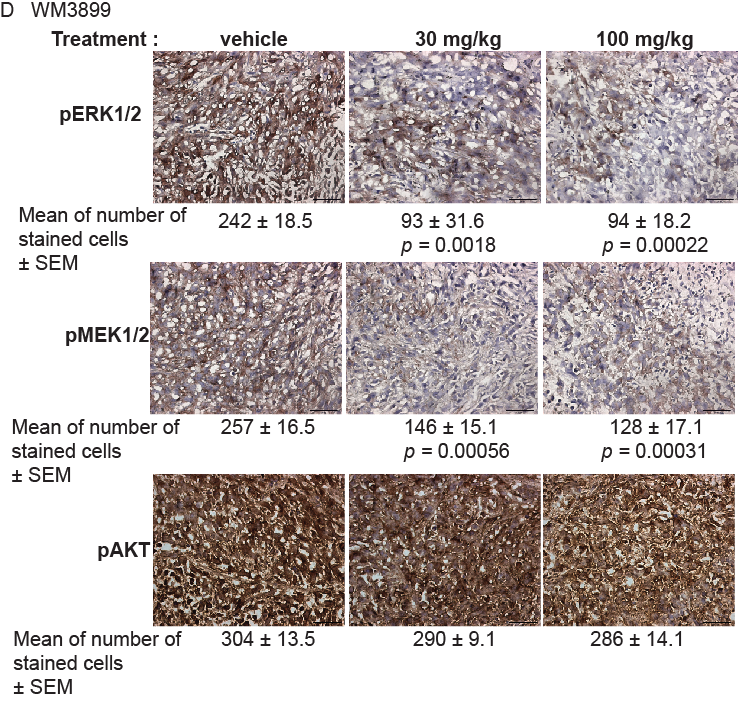

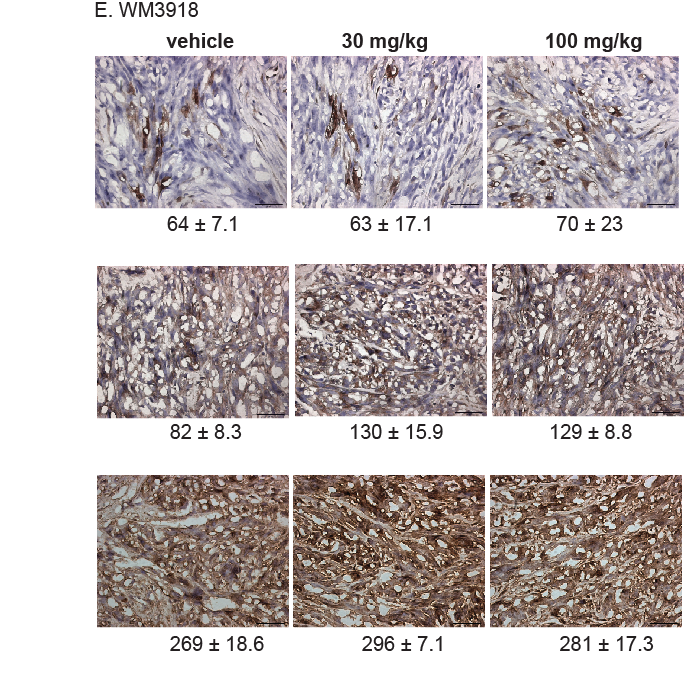

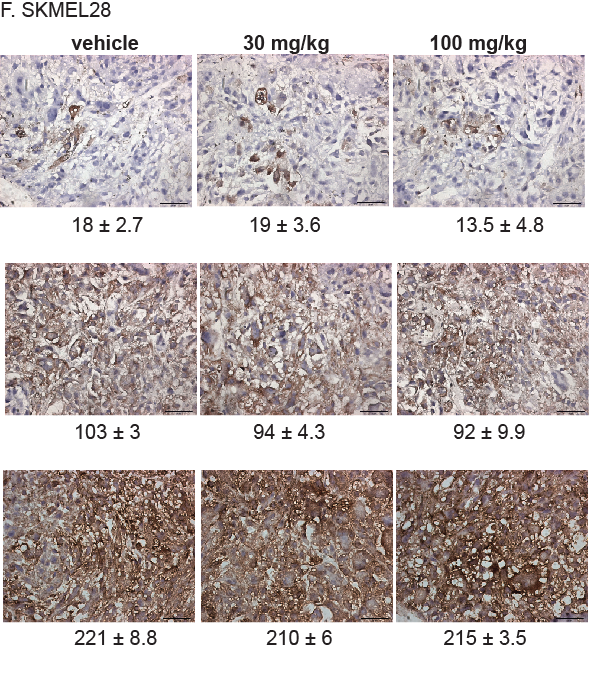
**


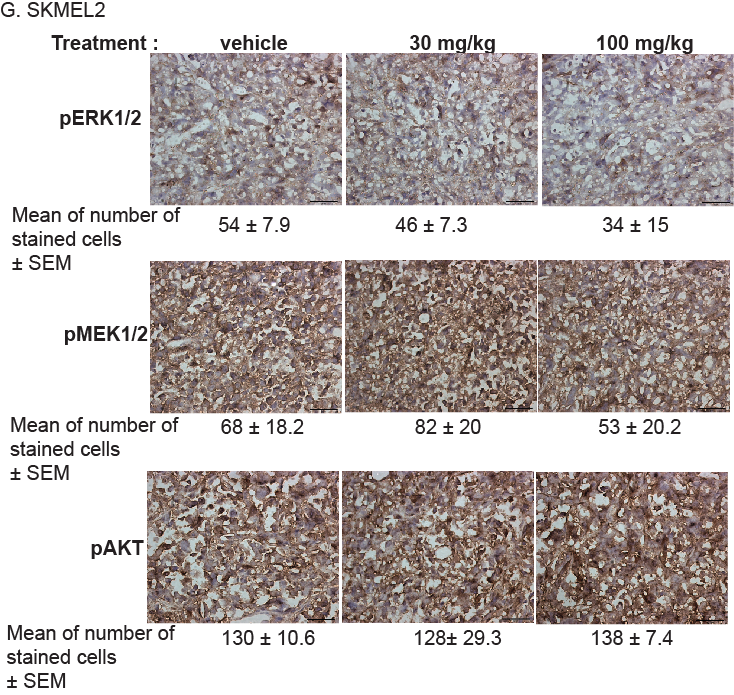


**Figure S3. pERK1/2, pMEK1/2 and pAKT staining in primary tumors.** For each cell line, pERK1/2, pMEK1/2 and pAKT expression was quantified on five mice per treatment group. Three photographs of “hot spot” staining per section were used to quantify the number of positive cells. P values are shown for the markers that achieved significance at a given dose of pazopanib (P < 0.01).
