# Supplementary material for: The B-Raf Status of Tumor Cells May Be a Significant Determinant of Both Antitumor and Anti-Angiogenic Effects of Pazopanib in Xenograft Tumor Models
Source: PLoS One. 2011 Oct 5;6(10):e25625. doi: 10.1371/journal.pone.0025625 (PMC3187787; doi:10.1371/journal.pone.0025625)
Supplement: Figure S4 — CD31 staining in primary tumors. Five mice per group and one section per mouse were stained for CD31. Three photographs of “hot spot” staining were used for quantification. Panels A and B show representative photographs of CD31 staining for each tumor (100× magnification). The AxioVision4 software was used to quantify the number of blood vessels per photograph and the percentage of area occupied by blood vessels, (numbers under each photograph in A and B). The numbers represent the mean number of vessels ± SEM in three “hot spots” per section. P values are shown for the markers that achieved significance at a given dose of pazopanib (P<0.01). (DOC) [file pone.0025625.s004.doc]

**
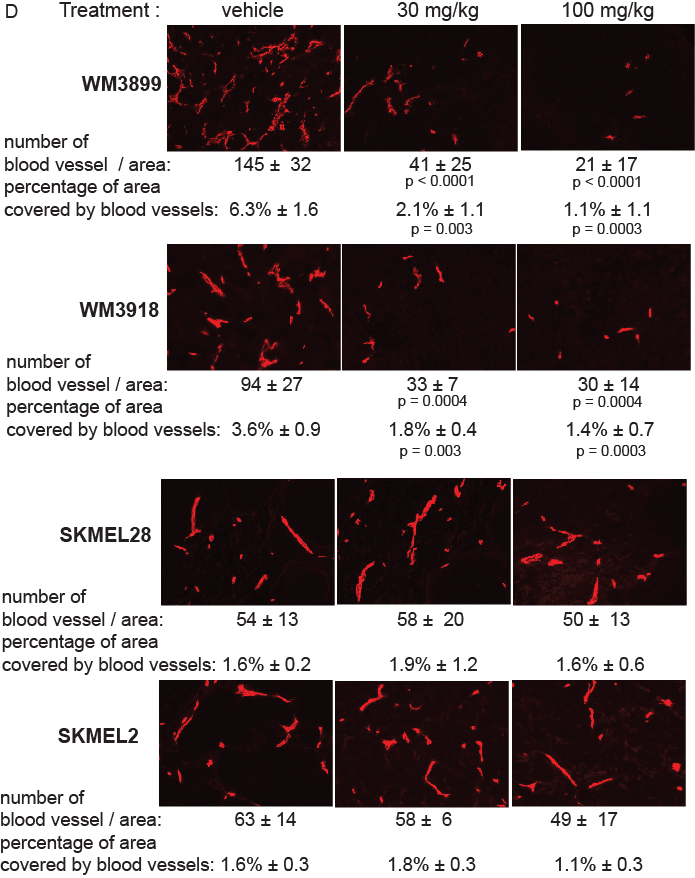
**


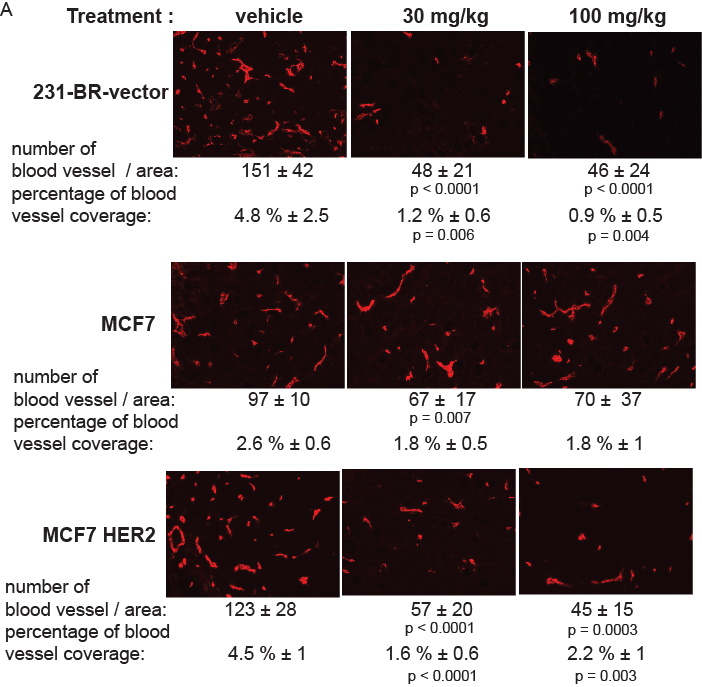


**Figure S4. CD31 staining in primary tumors.** Five mice per group and one section per mouse were stained for CD31. Three photographs of “hot spot” staining were used for quantification. Panels A and B show representative photographs of CD31 staining for each tumor (100x magnification). The AxioVision4 software was used to quantify the number of blood vessels per photograph and the percentage of area occupied by blood vessels, (numbers under each photograph in A and B). The numbers represent the mean number of vessels ± SEM in three “hot spots” per section. P values are shown for the markers that achieved significance at a given dose of pazopanib (P < 0.01).
