# Supplementary material for: The B-Raf Status of Tumor Cells May Be a Significant Determinant of Both Antitumor and Anti-Angiogenic Effects of Pazopanib in Xenograft Tumor Models
Source: PLoS One. 2011 Oct 5;6(10):e25625. doi: 10.1371/journal.pone.0025625 (PMC3187787; doi:10.1371/journal.pone.0025625)
Supplement: Figure S5 — B-Raf siRNA transfection in the 231-BR and the MCF7-HER2 cell lines. 231-BR (A) and MCF7-HER2 (B) cell lines were transfected with two different B-Raf siRNA constructs (S1 and S2), with a non targeting siRNA (C), or treated with the transfection agent alone (T). Cell lysates were collected at 48, 72 and 96 h after transfection and analyzed by western blot for B-Raf, Tubulin, PlGF and VEGF expression levels. (PDF) [file pone.0025625.s005.pdf]

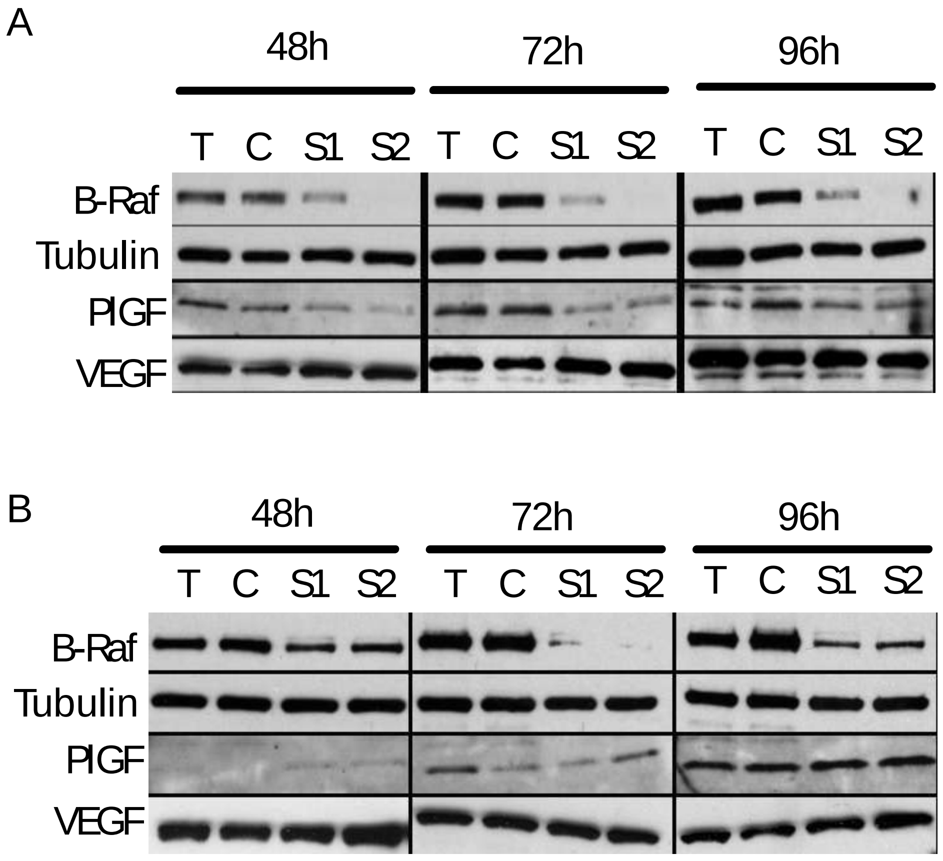

Figure S5. B-Raf siRNA transfection in the 231-BR and the MCF7-HER2 cell lines. 231-BR (A) and MCF7-HER2 (B) cell lines were transfected with two different B-Raf siRNA constructs (S1 and S2), with a non targeting siRNA (C), or treated with the transfection agent alone (T). Cell lysates were collected at 48, 72 and 96h after transfection and analyzed by western blot for B-Raf, Tubulin, PlGF and VEGF expression levels.
