# Supplementary material for: The B-Raf Status of Tumor Cells May Be a Significant Determinant of Both Antitumor and Anti-Angiogenic Effects of Pazopanib in Xenograft Tumor Models
Source: PLoS One. 2011 Oct 5;6(10):e25625. doi: 10.1371/journal.pone.0025625 (PMC3187787; doi:10.1371/journal.pone.0025625)
Supplement: Table S1 — In vitro growth inhibition of pazopanib on breast carcinoma and melanoma cell lines by MTT assay. (DOC) [file pone.0025625.s006.doc]

Table S1. In vitro growth inhibition of pazopanib on breast carcinoma and melanoma cell lines by MTT assay.

| **Cell Lines** | **B-Raf Status** | **Ras Status** | **Pazopanib IC50 (µM)** ^†^ |
| --- | --- | --- | --- |
| 231-BR | G464V | G13D (K-Ras) | 5 |
| MCF7-HER2 | WT | WT | 7 |
| MCF7 | WT | WT | 9.5 |
| WM3899 | G469V | WT | 4 |
| WM3918 | WT | WT | 4 |
| SKMEL28 | V600E | WT | 9 |
| SKMEL2 | WT | Q61R (N-Ras) | 8 |

^†^ IC_50_ measured using MTT cell viability assay 96h after pazopanib treatment.
