# Supplementary material for: The B-Raf Status of Tumor Cells May Be a Significant Determinant of Both Antitumor and Anti-Angiogenic Effects of Pazopanib in Xenograft Tumor Models
Source: PLoS One. 2011 Oct 5;6(10):e25625. doi: 10.1371/journal.pone.0025625 (PMC3187787; doi:10.1371/journal.pone.0025625)
Supplement: Material and Methods S1 — B-Raf kinase assay. (DOC) [file pone.0025625.s007.doc]

**Supplementary Material and Methods S1**

**B-Raf kinase assay.** Increasing concentrations of pazopanib were incubated with 50 µg of 231-BR, MCF7-HER2, MCF7, SKMEL2, SKMEL28, WM3918 or WM3899 cell lysate. ATP-Mg cocktail (20 µL) and Assay Dilution Buffer I (22 µL) were added to the samples and incubated for 20 min at 30 °C. Subsequently, inactive MEK1 was added to each mixture and incubated for 30 min at 30 °C. Twenty microliters of each reaction was used to perform pMEK1 and total MEK1 immunoblot analysis. The reagents were obtained from Upstate Biotechnology. Two independent experiments were performed.
