# Supplementary material for: The B-Raf Status of Tumor Cells May Be a Significant Determinant of Both Antitumor and Anti-Angiogenic Effects of Pazopanib in Xenograft Tumor Models
Source: PLoS One. 2011 Oct 5;6(10):e25625. doi: 10.1371/journal.pone.0025625 (PMC3187787; doi:10.1371/journal.pone.0025625)
Supplement: Material and Methods S2 — B-Raf siRNA transfection followed by cell viability assay. (DOC) [file pone.0025625.s008.doc]

**Supplementary Material and Methods S2**

**B-Raf siRNA transfection followed by cell viability assay.** 1.5 x 10^6^ cells were treated with 150 pmol siRNA and RNAiMax (Invitrogen). After 24h, cells were trypsinized and plated in 96-well plates at 2000 cells/well. The next day cells were treated with increasing concentrations of pazopanib (0.5, 1, 2, 4, 6, 8 and 10 µM) or with DMSO as a control, for 96 hours. Cells were lysed at 48 and 144 hours after transfection, which corresponded to the beginning of pazopanib treatment and the measure of cell viability by MTT, respectively. The number of viable cells was determined by adding 3-(4,5-dimethyl-2-thiazolyl)-2,5-diphenyl-2H-tetrazolium bromide (MTT; Sigma) at a final concentration of 0.5 mg/mL. Following incubation, MTT was dissolved in DMSO and absorbance was measured at 570 nm. Results are representative of three independent experiments, with each sample performed in sextuplicate.
