# Supplementary material for: The B-Raf Status of Tumor Cells May Be a Significant Determinant of Both Antitumor and Anti-Angiogenic Effects of Pazopanib in Xenograft Tumor Models
Source: PLoS One. 2011 Oct 5;6(10):e25625. doi: 10.1371/journal.pone.0025625 (PMC3187787; doi:10.1371/journal.pone.0025625)
Supplement: Material and Methods S3 — DCE-MRI Analysis. (DOC) [file pone.0025625.s009.doc]

**Supplementary Material and Methods S3**

**DCE-MRI Analysis.** Dynamic MR images were fitted to the two-compartment Generalized Kinetic Model (GKM) using a code written in IDL to calculate maps of the pharmacokinetic parameters for contrast uptake, Ktrans, and washout, Kep. In this analysis, the pre-contrast T1 map calculated from the low and high FA images were used to convert MR intensities to Gd concentration. Region of interests (ROI) were manually drawn around each tumor on the T2w image slice through the middle of the tumor and then copied to the DCE parametric maps to measure the average value, tumor size, and histogram in the tumor. The average rate constants were compared between the untreated and treated groups. The normalized cumulative histograms were also computed and averaged for each group and computed using Students T-test statistic on a point by point basis to determine subtle changes in the distribution of pharmacokinetic parameters with treatment.
