# Supplementary material for: The B-Raf Status of Tumor Cells May Be a Significant Determinant of Both Antitumor and Anti-Angiogenic Effects of Pazopanib in Xenograft Tumor Models
Source: PLoS One. 2011 Oct 5;6(10):e25625. doi: 10.1371/journal.pone.0025625 (PMC3187787; doi:10.1371/journal.pone.0025625)
Supplement: Material and Methods S4 — Statistical analysis. (DOC) [file pone.0025625.s010.doc]

**Supplementary Material and Methods S4**

**Statistical analysis.** A variety of analyses of variances (ANOVA) were performed on the raw or transformed data. Samples from experimental units were averaged and the square root of the sample size was used at a weight in subsequent analyses. One-way or two-factor factorial ANOVAs were used to analyze relatively simple fixed effect models. Mixed models were used as necessary to model random variation due to repeated experiments. Repeated measures ANOVAs were used to model longitudinal data. Analysis of covariance was used to account for important covariates. Binomial data was analyzed with a logit function. Non-linear regression (y=ae^xb^) was used to estimate decay rates (b) and IC50s were then calculated by letting y=50 and solving for x. If necessary, data were square root or cube root transformed to normalize residuals. Finally, for all ANOVAs, residuals were examined for normality and homogeneity and residuals were partitioned if found to be heterogeneous. For pair-wise comparisons between a control and other treatments, Dunnett’s method was used to adjust the p-values. Otherwise, Holm’s method was used to adjust pair-wise p-values [1]. In view of the multiple tests performed, we consider p<0.01 as the upper limit of what may be interpreted as being statistically significant, 0.01 < p <0.05 as a strong trend, and p>0.05 as not statistically significant.

**Reference**

1. Holm S (1979) A Simple Sequentially Rejective Multiple Test Procedure. Scandanavian Journal of Statistics 6: 65 -70.
